# Celastrol Ameliorates Vincristine-induced Neuropathic Pain by Inhibiting Spinal Astrocyte Hyperactivation-mediated Inflammation, Oxidative Stress, and Apoptosis

**DOI:** 10.2174/011570159X385690250509050208

**Published:** 2025-05-14

**Authors:** Gui-Zhou Li, Jing Xu, Yun-Man Li, Ya-Hui Hu

**Affiliations:** 1 Ministry of Education Key Laboratory of Model Animal for Disease Study, Model Animal Research Center, Jiangsu Key Laboratory of Molecular Medicine, Medical School, Nanjing University, Nanjing 210032, China;; 2 Pharmaceutical Sciences Research Center, Department of Pharmacy, Children’s Hospital of Nanjing Medical University, Nanjing 210008, China;; 3 School of Basic Medicine and Clinical Pharmacy, China Pharmaceutical University, 24 Tongjiaxiang, Nanjing 210009, China

**Keywords:** Celastrol, VINP, astrocyte, inflammation, oxidative stress, apoptosis

## Abstract

**Background:**

Neurotoxicity is the severe adverse reaction induced by chemotherapy drugs, characterized by neuropathic pain. However, there is a notable lack of therapeutic drugs for chemotherapy-induced neuropathic pain (CINP). Celastrol, a naturally occurring terpenoid active compound extracted from the roots of *Tripterygium wilfordii* Hook f., exhibits a neuroprotective effect, yet its therapeutic potential in CINP has not been reported.

**Objective:**

In this study, with vincristine-induced neuropathic pain (VINP) as a model, we aimed to investigate the therapeutic effect of celastrol on VINP and its specific mechanisms.

**Methods:**

Vincristine (VCR, 0.1 mg/kg, intraperitoneal injection) was used to induce a neuropathic pain model. Celastrol (0.5, 1.0, and 2.0 mg/kg, intraperitoneal injection) was administered to assess its therapeutic effects on VINP. Transmission electron microscopy (TEM) was employed to examine damage to the sciatic nerve fibers and mitochondria. Flow cytometry was used to detect mitochondrial membrane potential (MMP), reactive oxygen species (ROS), and cell apoptosis. Primary astrocyte cultures were utilized further to validate the therapeutic mechanisms of celastrol in VINP.

**Results:**

Here, we demonstrate that celastrol inhibits VCR-induced activation of spinal astrocytes by suppressing CaMKII phosphorylation. Additionally, celastrol alleviates the Cx43-dependent inflammation caused by VCR through the inhibition of the CaMKII/NF-κB signaling pathway. Concurrently, celastrol modulates the production of reactive oxygen species (ROS) and the expression of apoptosis-related proteins (Cleaved Caspase-3, Bax, and Bcl-2) by suppressing the phosphorylation of CaMKII in astrocytes, thereby ameliorating the mitochondrial damage and cell apoptosis caused by VCR.

**Discussion:**

This study delves into the efficacy of celastrol in treating VINP and elucidates its underlying mechanisms. The findings demonstrate that celastrol elevates pain thresholds in mice, ameliorates neuropathy, and inhibits VCR-induced astrocyte activation, as well as spinal dorsal horn inflammation, oxidative stress, and apoptosis, by blocking CaMKII phosphorylation. Unlike first-line CINP drugs, celastrol targets multiple CINP-related pathological pathways. However, this study primarily focuses on male mice and lacks a naive group, which may affect the interpretation of baseline physiological parameters. Therefore, future research will incorporate female mice and naive groups to further enhance the study's comprehensiveness and reliability.

**Conclusion:**

Our findings reveal that celastrol exerts therapeutic effects on VINP through its anti-inflammatory, antioxidant, and anti-apoptotic properties. Furthermore, we preliminarily explore the molecular mechanisms underlying these effects, thereby providing a scientific basis for celastrol as a potential therapeutic agent for CINP.

## 1 INTRODUCTION

At present, chemotherapy remains one of the primary therapeutic approaches for cancer. However, chemotherapeutic drugs such as taxanes and platinum-based agents can directly damage the peripheral nervous system, leading to peripheral neuropathy [1, 2]. This condition is characterized by symmetrical pain, numbness, and altered touch sensation in the extremities. It severely impacts patients' adherence to treatment regimens and diminishes their quality of life. Vinca alkaloids, exemplified by vincristine, also exhibit neurotoxic properties [3, 4]. Their active constituents can disrupt axonal microtubules and interfere with transport mechanisms, which manifests in symptoms such as paresthesia, dysesthesia, and an increased risk of developing multiple peripheral neuropathies [5]. In the context of vincristine-induced neuropathic pain (VINP), the pronounced activation of glial cells suggests that central sensitization may be a contributing mechanism to pain genesis, potentially associated with an upregulation of the release of inflammatory mediators by these cells [6]. Our recent study also found that CaMKII and Cav3.2 T-type calcium channels mediate Cx43-dependent inflammation by activating astrocytes in VINP [7].

The management of chemotherapy-induced neuropathic pain (CINP) primarily relies on dose reduction or discontinuation of the chemotherapeutic agents responsible for inducing CINP, complemented by symptomatic treatment for neuropathic pain [5, 8]. Fifteen clinical trials have been conducted to investigate the prevention and symptomatic management of CINP, with only duloxetine demonstrating efficacy in treating established chemotherapy-related neuropathic pain [9]. While the introduction of newer chemotherapeutic drugs offers hope for a reduction in CINP, many older agents that induce neuropathic pain remain indispensable in cancer therapy [10, 11]. Additionally, a number of newer agents also present CINP as a dose-limiting side effect [12, 13]. Therefore, a deeper understanding of the pathophysiological mechanisms of CINP and the development of effective therapeutics for CINP are of paramount importance for improving chemotherapy compliance and alleviating patient suffering.

Celastrol, a naturally occurring triterpenoid compound extracted from the traditional Chinese medicinal plant *Tripterygium wilfordii*, has been demonstrated to possess significant anti-inflammatory and antioxidant capabilities [14-16]. In recent years, its role in neuroprotection has also been progressively validated [17-20]. Celastrol reduces neuroinflammatory response and inhibits oxidative stress and apoptosis of neurons, thereby exerting neuroprotective effects [17-20]. Consequently, celastrol has exhibited significant activity in neuroprotection, anti-inflammation, and antioxidant stress, providing a scientific rationale for its application in the treatment of neuropathic pain. Indeed, our research revealed that celastrol significantly ameliorates inflammation, oxidative stress, and apoptosis caused by the overactivation of spinal dorsal horn astrocytes due to VCR, thus playing a therapeutic role in neuropathic pain. These findings highlight the potential clinical application value of celastrol in the treatment of CINP.

## 2 MATERIALS AND METHODS

### 2.1 Animals

A total of 160 adult male ICR mice (8 weeks old, weighing 25-30 g) and 40 adult female ICR mice (8 weeks old, weighing 20-25 g) were sourced from Jackson Laboratory (Shanghai, China). The animals were maintained under a standardized 12-hour light/dark cycle with ambient temperature and relative humidity meticulously controlled at 22-25°C and 60 ± 10%, respectively. All animal studies were carried out strictly in line with the ARRIVE guidelines and conformed to the policies of the International Association for the Study of Pain. Moreover, all animal studies were performed strictly following the institutional guidelines for the humane treatment of animals and were approved by The Institutional Animal Care and Use Committee of the Model Animal Research Center of Nanjing University, China. The associated approval number is AP#SY17.

### 2.2 Chemotherapy-related Pain Induction and Drug Administration Protocols

Mice received daily intraperitoneal injections of vincristine sulfate (VCR, 0.1 mg/kg; dissolved in physiological saline) for five consecutive days, procured from Shenzhen Main Luck Pharmaceutical Incorporated, China (injection volume: 0.1 ml per 10 g body weight). Control animals were administered an equivalent volume of physiological saline. Celastrol (MedChemExpress, HY-13067) was formulated in a vehicle solution to achieve the desired concentration for intraperitoneal administration, which was initiated on the first day following the final VCR injection and continued for seven days. Concurrently, KN-93 (70 nM, 10 μl; Selleck, S7423) and Gap27 (144 nM, 10 μl; MedChemExpress, HY-P0139) were administered intrathecally into the dorsal subarachnoid space of the mice daily for two consecutive days, commencing post-VCR injection. Sham-operated animals received an equivalent volume of the vehicle solution intrathecally. Notably, these compounds were administered independently following VCR modeling, without administering two or more drugs simultaneously.

### 2.3 Behavioral Assessments of Mechanical Allodynia and Heat Hyperalgesia

This study assessed mechanical hypersensitivity and thermal hyperalgesia in mice under consistent, quiet environmental conditions within a designated testing room. Mechanical allodynia was evaluated using a range of Von Frey filaments (0.07 to 2.0 g) to ascertain the mice's mechanical pain threshold. Mice were initially acclimated in a transparent plexiglass cage placed atop an elevated metal mesh for 30 min prior to testing. Once the mice were calm, each Von Frey filament was applied vertically to the plantar surface of the hind paw five times in ascending order, with each application lasting 2-3s. The testing was discontinued when three out of five applications with the filaments elicited a withdrawal response, such as licking or lifting the paw. The 50% paw withdrawal threshold (PWT) was computed *via* Dixon's up-down statistical method. Thermal hyperalgesia was tested by gauging the latency of paw withdrawal in reaction to thermal radiation with a PL200 thermal radiometer. As per established protocols, the intensity of the radiant heat was calibrated to keep the paw withdrawal latency (PWL) of the sham-operated group within 10 ± 2s.

### 2.4 Paraffin and Frozen Sections

The mice were anesthetized with isoflurane and initially perfused *via* the ascending aorta with saline. Once the effluent was clear of blood, the saline was replaced with 4% paraformaldehyde for perfusion. The mice were then placed on an ice board, and the T2-L2 spine was extracted and placed in 4% paraformaldehyde for 24 h. The spinal lamina was carefully removed with forceps, and the L1-L6 spinal cord tissue was extracted and fixed in 4% paraformaldehyde for 24 h. A portion of the fixed spinal cord tissue was dehydrated using a dehydrator (for paraffin sectioning), while another portion was placed in 25% and 30% sucrose solutions for gradient dehydration (for frozen sectioning). After dehydration, the spinal cord tissue dehydrated by the dehydrator was embedded in paraffin, and the embedded tissue was sliced using a paraffin microtome to a thickness of 5 μm. The sucrose gradient-dehydrated spinal cord tissue was embedded with OTC glue, placed into a cryostat for pre-freezing, and then sliced to a thickness of 25 μm using the cryostat. The cut tissue sections were washed with PBS, transferred to a PBS solution containing 50% glycerol, and stored at –20°C.

### 2.5 Transmission Electron Microscopy (TEM)

The sciatic nerve tissue was rapidly removed and soaked in a primary fixation solution consisting of 2.0% glutaraldehyde (a grade suitable for electron microscopy) and 2.5% formaldehyde. The tissue was then cut into 1 mm^3^ cubes. The cubes were then placed in the above primary fixings, held at room temperature for 30 min, and then extended overnight at 4°C. Subsequent TEM sample preparation and imaging were performed by Shandong Weiya Biotechnology Co., LTD., following the TEM analysis standard protocol. Specifically, after primary fixation, the tissue cubes were subjected to secondary fixation with 2% osmium tetroxide in 0.1 M phosphate buffer (pH 7.4) for 2 h at room temperature. They were then dehydrated through a graded series of ethanol solutions (30%, 50%, 70%, 80%, 90%, 95%, and 100%) for 15 min at each concentration, embedded in epoxy resin, and polymerized at 60°C for 24-48 hours. Ultrathin sections (70-90 nm) were cut using an ultramicrotome (Leica EM UC7) and collected on copper grids, then stained with uranyl acetate and lead citrate. TEM imaging was performed using a (JEOL JEM-1400) transmission electron microscope operated at an acceleration voltage of (80 kV), equipped with a charge-coupled device (CCD) camera (Gatan Ultrascan 4000) with a resolution of (4096 × 4096 pixels) to capture high-quality images at various magnifications. Image analysis and quantitative assessments were conducted using ImageJ 1.51 (Rawak Software Inc., Stuttgart, Germany), measuring parameters such as organelle size (*via* outlining and the ROI manager) and number (*via* manual counting and density calculation) and other structural parameters (*via* appropriate software tools). The quantitative data were analyzed using statistical software (GraphPad Prism8) for descriptive statistics and visualized using graphs and charts.

### 2.6 Primary Astrocyte Cultures

The procedures for culturing primary astrocytes have been documented previously [5]. To elaborate, the cerebral cortex from neonatal ICR mice was extracted under sterile conditions. Following the meticulous removal of the meninges, the cortical tissue was diced into small fragments and subjected to digestion with papain (2 mg/mL) for 30 min. Subsequently, the cell suspension was processed by centrifugation, filtered, and re-suspended in DMEM/F-12 medium (Wisent, Canada) enriched with 10% (v/v) fetal bovine serum (FBS) (AusGeneX, Molendinar, Australia). Cultivation of the cells takes place in a humidified incubator maintained at 37°C with 5% CO_2_, with medium changes occurring bi-weekly. Once the cells achieved 90% confluence, astrocyte purification was accomplished by agitating the culture flask at around 200 rpm for 4 h. The astrocyte purity was verified *via* glial fibrillary acidic protein (GFAP) staining, yielding a positivity rate of 85-95%, and the cells displayed a typical star-like morphology. The cells were pre-incubated with celastrol (1.0 μM or 2.0 μM) or KN-93 (10 μM) for 6 h, followed by a 24-hour exposure with VCR (3 nM) (3 nM).

### 2.7 Hematoxylin-Eosin (HE) Staining

Mouse sciatic nerves were prepared for histological examination by overnight fixation in tissue fixative, followed by dehydration and paraffin embedding. Sections were cut to a thickness of 5 μm using a microtome. The sections underwent standard deparaffinization and hydration processes before being stained with hematoxylin for 1 min. Tissue differentiation was achieved with a 1% hydrochloric acid ethanol solution for 2 seconds, followed by a 1 min rinse in running water. Subsequent staining with 1% water-soluble eosin was performed for approximately 3 min. After a final 1 min rinse, tissues were dehydrated through a graded series of ethanol and cleared with xylene. The sciatic nerve samples were then examined for pathological changes under an inverted microscope, and the morphological alterations were documented and analyzed.

### 2.8 Immunofluorescence Analysis

Sections of the L4-L5 spinal cord, measuring 25 μm in thickness, were generated using a cryostat (Thermo Fisher Scientific, Waltham, USA) and preserved in a solution containing 50% glycerol at −20°C prior to immunofluorescence staining. To summarize, these sections were initially blocked with a buffer composed of 0.3% Triton X-100 and 5% goat serum in phosphate buffer saline (PBS) for 30 min. Subsequently, they were incubated with primary antibodies: p-CaMKII (ThermoFisher, MA1-047, diluted to 1:100) and GFAP (Cell Signaling Technology, 3670T, diluted to 1:300) overnight at 4°C, followed by three PBS washes. The sections were then treated with secondary antibodies conjugated to Cy3 (Bioss; AH06141740, 1:100) or FITC (Beyotime; A0568, 1:100) for 2 hours. Nuclei were stained using DAPI (KeyGEN Biotechnology, China). The stained sections were subsequently examined using a confocal microscope (Carl Zeiss, Germany). Image analysis and quantitative evaluation were conducted using ImageJ 1.51 software to assess parameters such as fluorescence intensity and occupied area.

### 2.9 Western Blot

Tissues from spinal segments (20 mg) were gathered for protein extraction using RIPA lysis buffer (Beyotime Biotech, Nantong, China), which included proteinase inhibitors (1 mM, Applygen Technologies, Beijing, China) and phosphatase inhibitors (1 mM, Applygen Technologies, Beijing, China). Cells were plated in 6-well plates, rinsed with PBS, and treated with 100 μl of RIPA lysis buffer containing proteinase inhibitors. Following 30 min of lysis on ice, the samples were centrifuged at 12000 rpm for 15 min. Equal amounts of protein (50 μg per lane) were loaded into SDS-PAGE gels for separation and transferred to PVDF membranes. Primary antibodies (1:1000) against phospho-CaMKII (p-CaMKII, ab171095) and CaMKII (ab134041) provided by Abcam; phospho-NF-κB P65 (p-P65, 3033T), NF-κB p65 (t-P65, 8242T), Bcl-2 (3498S), Bax (5023S), cleaved-Caspase-3 (661S), and Cx43 (3512S) brought from Cell Signalling Technology; Cav3.2 (PA5-72836, Thermo Fisher Scientific) were used to incubate blots. GAPDH (60004-1-Ig, 1:5000, Proteintech) was used as a loading control. Afterward, membranes were incubated with appropriate secondary antibodies (Proteintech; SA00001-1, 1:2000 or Beyotime; A0208, 1:2000) for 2 h. Protein bands were detected using an enhanced chemiluminescence system, and their intensities were quantified using densitometric analysis with ImageJ 1.51 software. Subsequently, the gray value of each target protein was normalized to its corresponding GAPDH gray value.

### 2.10 qRT-PCR

Total RNA extraction from L4-L5 spinal segments (10 mg) and cellular samples was isolated with TRIzol Reagent (9109, Takara) and then transcribed using the HiScript III RT SuperMix (R323, Vazyme). For qPCR, a ChamQ SYBR qPCR Master Mix (Q311, Vazyme) was employed. The comparative mRNA expression levels of the genes of interest were calculated with the 2^−ΔΔCT^ method, using GAPDH as the internal control. Table **1** provides a comprehensive list of primers used in the study.

### 2.11 ELISA

We employed ELISA kits (Dakewe Biotech, Beijing, China) to quantify the concentrations of IL-6 and IL-1β in serum, adhering to the manufacturers' guidelines for the assays.

### 2.12 Cell Viability Assay

The CCK-8 kit (KeyGen Biotechnology, China) was used to test cell viability. Primary astrocytes cultured in 96-well plates were exposed to a gradient of celastrol concentrations, ranging from 0 to 10 μM, over a 24 h period. Subsequently, 10 μl of the CCK-8 solution was introduced to each well for 2 h incubation. The resulting absorbance, measured at 450 nm, was quantified using a Multiskan FC microplate photometer from ThermoFisher Scientific.

### 2.13 Measurement of Intracellular Free Calcium Level

Intracellular free calcium levels ([Ca^2+^]_i_) were measured using the Fluo-4 AM (calcium-sensitive dye, Beyotime, Shanghai). Astrocyte cultures were gently washed with cold, sterile Hank's balanced salt solution (HBSS), subsequently loaded with 5 μM Fluo-4 AM, and incubated at 37°C for 45 minutes. After incubation, cells were washed with HEPES buffer solution composed of (in mM) 5 KCl, 137 NaCl, 10 HEPES, 0.5 MgCl_2_, 1 CaCl_2_, 1 Na_2_HPO_4_, supplemented with 1% bovine serum albumin (BSA) and 10 glucose, adjusted to pH 7.4 The cells were then further incubated in HBSS containing 1% serum for 40 min before fluorescence intensity was assessed using a confocal microscope.

### 2.14 Assessment of MMP and ROS

Astrocyte mitochondrial membrane potential (ΔΨm) and total reactive oxygen species (ROS) levels were determined using specialized assay kits. The mitochondrial membrane potential (MMP) was assessed with a JC-1-based mitochondrial membrane potential assay kit, while the ROS levels were measured with a ROS species assay kit, both kits from Beyotime. After performing the assays according to the manufacturer's instructions, the samples were analyzed using flow cytometry (BD Biosciences, CA, USA) to capture the fluorescence changes indicative of MMP and ROS levels.

### 2.15 Apoptosis Assessment *via* Annexin V/PI Staining

Following the supplier’s guidelines, astrocytes were thoroughly rinsed with PBS three times and subsequently subjected to double staining with an Annexin V-FITC Apoptosis Detection Kit (BD Biosciences, CA, USA). The staining procedure was conducted for a duration of 15 min at ambient temperature in a dark environment to prevent photobleaching. The stained cells indicating apoptosis were analyzed using a flow cytometer.

### 2.16 Statistical Analysis

In our analyses, mean values were compared using unpaired two-tailed Student’s *t*-tests for two-group comparisons, and one-way or two-way ANOVA for multiple-group comparisons, followed by Bonferroni post hoc tests where appropriate. Statistical significance was set at a *p*-value threshold of less than 0.05, with asterisks (*, **, and ***) indicating *p*-values of less than 0.05, 0.01, and 0.001, respectively. Data visualizations presented mean values accompanied by the standard error of the mean (SEM), as detailed in the figure legends. For data analysis, we relied on SPSS 22.0 (IBM, Armonk, USA), and for graphing, GraphPad Prism software version 8.0.1 (GraphPad Software, CA, USA) was employed.

## 3 RESULTS

### 3.1 Celastrol Alleviates VINP and Sciatic Nerve Injury in Mice

**Fig. (1):**
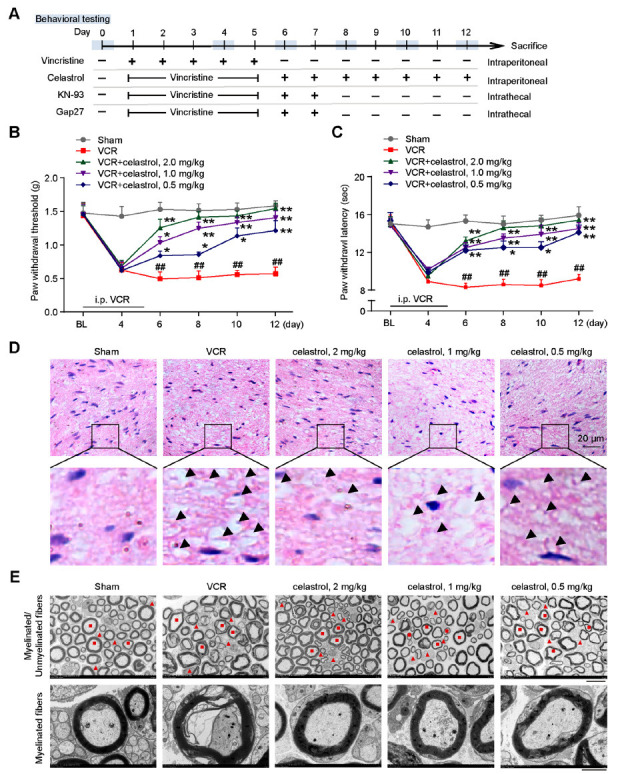
Celastrol alleviates VINP and sciatic nerve injury in mice. (**A**) The experimental paradigms of *in vivo* analysis. Celastrol attenuated mechanical (**B**) and thermal hyperalgesia (**C**) in male mice with VINP. Celastrol (0.5, 1 and 2 mg/kg, i.p.) was administered after the VCR (0.1 mg/kg) intraperitoneal injection. *n* = 8 mice per group. (**D**) Representative H&E staining of sciatic nerve. scale bar = 20 μm, *n* = 6 mice per group. (**E**) Representative electron micrographs analysis of the sciatic nerve. SC, Schwann cell; red ■, myelinated nerve fibers; red▲, Unmyelinated nerve fibers. scale bar = 10 μm or 2 μm, *n* = 4 mice per group. Data are presented as mean ± SEM. Sham *vs.* VCR: **^##^**; VCR *vs.* VCR + celastrol: *. **^#^***P* < 0.05, **^##^***P* < 0.01, and **^###^***P* < 0.001. *P < 0.05, **P < 0.01, and ***P < 0.001. Two-way ANOVA tests were performed in **B** and **C**.

Experimental paradigms were graphically represented in Fig. **1A**. Mice were administered VCR intraperitoneally daily for five consecutive days to establish a model of VINP. Beginning on the sixth day of post-modeling and continuing through the twelfth day, celastrol was administered intraperitoneally at dosages of 0.5, 1.0, and 2.0 mg/kg. Following the initial dose of celastrol in the three tested dosage groups, a significant amelioration of mechanical allodynia and thermal hyperalgesia in male mice was observed compared with the VCR model group (Figs. **1B** and **C**). Additionally, as the number of celastrol administrations increased, the PWT and PWL in the medium and high-dose groups demonstrated a progressive improvement, ultimately approximating the values shown in the sham group (Figs. **1B** and **C**). However, the treatment effects of celastrol on VINP in female mice were only observed in the high-dose group (Figs. **S1A** and **B**). Given the significant inter-individual variability in the treatment response among female mice, the effects were less marked and stable compared with those observed in male mice, and our study primarily focused on male mice.

Polyneuropathy is a severe adverse effect associated with VCR therapy. To assess the neuroprotective impact of celastrol on VCR-induced sciatic nerve damage in mice, sciatic nerves were collected on the 12^th^ day following treatment for H&E staining. The VCR group displayed pronounced sciatic nerve degeneration, marked by a loosely organized structure of nerve fibers, variable fiber density, and axonal atrophy or loss, as indicated by arrows. In contrast, celastrol-administered groups at 0.5, 1.0, and 2.0 mg/kg showed less severe fiber disarray and density irregularities, with a noticeable reduction in vacuolar degeneration and edematous changes (Fig. **1D**). Furthermore, TEM analysis suggested that in the VCR model group, sciatic nerve cross-sections exhibited Schwann cell (SC) edema, severe damage to the myelin sheaths of myelinated nerve fibers (shown in red ■), localized demyelination. Unmyelinated nerve fibers (shown in red ▲) showed disrupted neurofilaments and microtubules, appearing as amorphous, fluffy material. Celastrol treatment ameliorated these pathological changes, in which the most considerable improvements were found in the high-dose group (Fig. **1E**). These data suggested that celastrol may ameliorate VINP by improving peripheral neuropathy.

### 3.2 Celastrol blocks VCR-induced Astrocytes Hyperactivation in the Spinal Cord Dorsal Horn

**Fig. (2):**
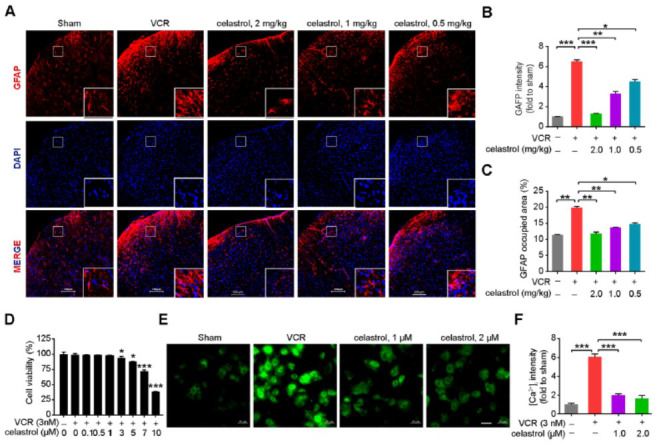
Celastrol blocks VCR-induced astrocyte hyperactivation. (**A**) Representative confocal images of GFAP (red), DAPI (blue), and quantitative analysis of (**B**) GFAP immunofluorescence intensity and (**C**) GFAP-occupied area in the spinal dorsal horn. scale bar = 100 μm, n = 5 mice/group. (**D**) Cell viability of astrocytes pre-incubated with varying concentrations of celastrol from 0.1 to 10 μM for 6 h, followed by a 24-hour exposure with VCR (3 nM). *n* = 6 cultures/group. (**E**) Representative [Ca^2+^]_i_ imaging and (**F**) quantitative analysis of immunofluorescence intensity in astrocytes. scale bar = 50 μm, *n* = 3 independent experiments. Data are presented as mean ± SEM and *P < 0.05, **P < 0.01, and ***P < 0.001. Unpaired two-tailed Student’s t-tests were performed in **B**-**D** and **F**.

Recent studies have revealed a marked escalation in astrocyte activation within the spinal cord of mice subjected to CINP [21, 22]. To elucidate the suppressive effect of celastrol on astrocyte activation in VCR-induced mouse spinal cord, immunofluorescence analysis was conducted on spinal cord samples obtained from mice on day 12. The findings indicated that, compared with the VCR model group, three different dosages of celastrol significantly reduced GFAP-positive fluorescence intensity, thereby markedly inhibiting astrocyte activation in the mouse spinal cord triggered by VCR (Figs. **2A**-**C**). Notably, the high-dose celastrol group (2.0 mg/kg) exhibited GFAP fluorescence intensity comparable to that of the sham group. Previous studies have indicated that Ca^2+^ signaling associated with astrocyte activation is involved in the regulation of CINP [7, 23, 24]. Subsequently, we isolated primary astrocytes and determined the concentration of celastrol (≤ 2 μM) that would not affect astrocyte viability (Fig. **2D** and Figs. **S2A** and **B**); we then assessed whether celastrol could ameliorate the impact of VCR on [Ca^2+^]_i_. The Ca^2+^ fluorescence imaging revealed that celastrol (1.0 μM and 2.0 μM) significantly alleviated the increase in [Ca^2+^]_i_ associated with VCR-induced astrocyte activation (Figs. **2E** and **F**). These results suggest that celastrol may alleviate the overactivation of spinal astrocytes in VCR-induced mice by reducing the [Ca^2+^]_i_ content associated with astrocyte activation.

### 3.3 Celastrol Mitigates VINP by Inhibiting CaMKII Activation

**Fig. (3):**
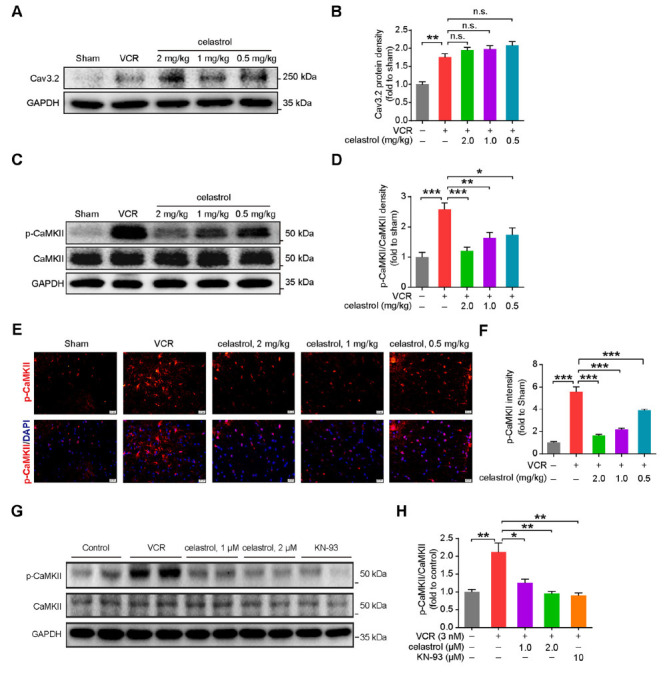
Celastrol mitigates VINP by inhibiting CaMKII activation. (**A**) Western blot and (**B**) quantitative analysis of Cav3.2 protein expression in the spinal cord from the indicated male mice. The three doses of celastrol showed no significant differences compared with the VCR group (*P* > 0.05) and were significantly higher than the Sham group (*P* < 0.005). *n =* 6 mice per group. (**C**) Western blot and (**D**) quantitative analysis of CaMKII and p-CaMKII protein expression in the spinal cord from the indicated male mice. The 2.0 mg/kg dose of celastrol exhibited a robust inhibitory effect on CaMKII, approaching that of the Sham group (*P* > 0.05). Although the 0.5 mg/kg and 1 mg/kg doses also had significant inhibitory effects, they still differed from the Sham group (*P* < 0.05). *n =* 6 mice per group. (**E**) Representative confocal images of p-CaMKII (red), DAPI (blue), and (**F**) quantitative analysis of p-CaMKII immunofluorescence intensity in the spinal cord. scale bar = 20 μm, *n* = 4 mice/group. (**G**) Western blot and (**H**) quantitative analysis of CaMKII and p-CaMKII proteins expression in astrocytes pre-incubated with celastrol (1.0 μM or 2.0 μM) and KN-93 (10 μM) for 6 h, followed by a 24-hour exposure with VCR (3 nM). *n* = 3 independent experiments. Data are presented as mean ± SEM and *P < 0.05, **P < 0.01, and ***P < 0.001. Unpaired two-tailed Student’s t tests were performed in **B**, **D**, **F**, and **H**.

In primary astrocytes, VCR leads to an increase in [Ca^2+^]_i_ by promoting the influx of Ca^2+^ from the extracellular fluid, which is regulated by Cav3.2, and the release of CaMKII-sensitive Ca^2+^ from intracellular stores [7]. Inhibition of Cav3.2 channel opening and reduction of CaMKII phosphorylation can ameliorate VINP [7]. We then examined the protein levels of Cav3.2 and CaMKII. Western blot results showed that none of the three doses of celastrol mitigated the increase in Cav3.2 protein levels in the spinal cord of mice with VINP (Figs. **3A** and **B**). This finding suggests that celastrol may not improve VINP by inhibiting the Ca^2+^ influx regulated by Cav3.2 Fortunately, both Western blot and immunofluorescence analyses revealed that the increased expression of phosphorylated CaMKII protein, observed in the VCR model group, was significantly reduced in a dose-dependent manner across the three treatment groups (Figs. **3C**-**F**). To further confirm the inhibitory effect of celastrol on CaMKII phosphorylation, we conducted additional validation in primary astrocytes cultured *in vitro*. We first treated primary astrocytes with celastrol (2 µM) to exclude the effect of celastrol on the phosphorylation of CaMKII in normal astrocytes (Fig. **S2C**). Then, we found that both doses of celastrol (1 µM and 2 µM) significantly inhibited the phosphorylation of CaMKII in astrocytes induced by VCR, with the higher dose showing efficacy comparable to that of the CaMKII phosphorylation-specific inhibitor KN-93 (10 µM). (Figs. **3G** and **H**). Therefore, we propose that the therapeutic effects of celastrol on VINP may be mediated by inhibiting the phosphorylation of CaMKII, reducing the release of intracellular stored calcium ions, thereby suppressing the activation of astrocytes.

### 3.4 Celastrol Ameliorates VCR-induced Inflammation by Suppressing the CaMKII/Cx43 Signaling Pathway

**Fig. (4):**
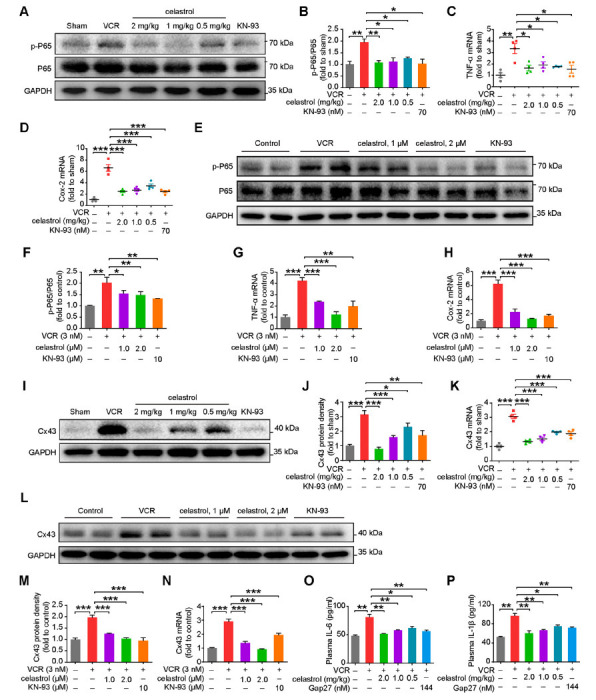
Celastrol ameliorates VCR-induced inflammation by suppressing the CaMKII/cx43 signaling pathway. (**A**) Western blot and (**B**) quantitative analysis of P65 and p-P65 protein expression in the spinal cord from the indicated male mice. *n =* 6 mice per group. (**C**, **D**) qRT-PCR analysis of spinal cord *TNF-α* and *Cox-2* mRNA levels, *n* = 4 mice/group. (**E**) Western blot and (**F**) quantitative analysis of P65 and p-P65 proteins expression in astrocytes pre-incubated with celastrol (1.0 μM or 2.0 μM) and KN-93 (10 μM) for 6 h, followed by a 24-hour exposure with VCR (3 nM). *n* = 3 independent experiments. (**G**, **H**) qRT-PCR analysis of primary astrocytes *TNF-α* and *Cox-2* mRNA levels, *n* = 3 independent experiments. (**I**) Western blot and (**J**) quantitative analysis of Cx43 protein expression in the spinal cord from the indicated male mice. *n =* 6 mice per group. (**K**) qRT-PCR analysis of spinal cord *Cx43* mRNA levels, *n* = 4 mice/group. (**L**) Western blot and (**M**) quantitative analysis of Cx43 protein expression in astrocytes pre-incubated with celastrol (1.0 μM or 2.0 μM) and KN-93 (10 μM) for 6 h, followed by a 24-hour exposure with VCR (3 nM). *n* = 3 independent experiments. (**N**) qRT-PCR analysis of *Cx43* mRNA levels in primary astrocytes pre-incubated with celastrol (1.0 μM or 2.0 μM) and KN-93 (10 μM) for 6 h, followed by a 24-hour exposure with VCR (3 nM), *n* = 3 independent experiments. (**O**, **P**) The levels of IL-6 and IL-1β in plasma were analyzed by ELISA. *n* = 6 mice/group. Data are presented as mean ± SEM and *P < 0.05, **P < 0.01, and ***P < 0.001. Unpaired two-tailed Student’s t-tests were performed in **B**-**D**, **F-H, J, K** and **M**-**P**.

Astrocyte activation leads to an increased release of inflammatory factors, which is a major cause of the development and progression of VINP [7]. CaMKII phosphorylation enhances the astrocyte-mediated production of inflammatory factors, which are subsequently released through Cx43, eliciting an inflammatory response within the spinal cord in VINP [7]. Therefore, we hypothesize that celastrol may modulate the inflammatory response by inhibiting the CaMKII signaling pathway, thereby treating VINP. The expression levels of proteins involved in the inflammatory response were evaluated in spinal cord tissues from mice. Western blot analysis revealed that celastrol, administered at three distinct dosages, and KN-93 significantly curtailed the expression of the phosphorylated p65 protein (Figs. **4A** and **B**). Additionally, qRT-PCR was utilized to quantify the mRNA levels of inflammation-related genes, tumor necrosis factor-alpha (TNF-α), and cyclooxygenase-2 (Cox-2), and revealed that both celastrol and KN-93 induced a downregulation of TNF-α and Cox-2 mRNA expression (Figs. **4C** and **D**). Similar results were observed in primary astrocytes cultured *in vitro*. (Figs. **4E**-**H**). Subsequently, we found that both celastrol and KN-93 effectively alleviated the upregulation of Cx43 expression induced by VCR in spinal cord tissues and primary astrocytes (Figs. **4I**-**N**). The use of celastrol and Gap27, a Cx43-specific inhibitor, significantly reduced the release of VCR-induced inflammatory factors in mouse plasma, including TNF-α and IL-1β (Figs. **4O** and **P**). Together, these observations underscore celastrol ameliorates Cx43-dependent inflammatory responses by inhibiting the activation of CaMKII.

### 3.5 Celastrol Improves VCR-induced Oxidative Stress and Mitochondrial Damage in Astrocytes

**Fig. (5):**
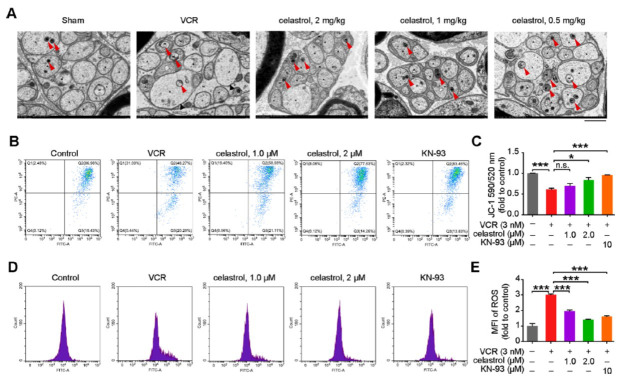
Celastrol improves VCR-induced oxidative stress and mitochondrial damage in astrocytes. (**A**) Representative electron micrograph analysis of the sciatic nerve from the indicated male mice. Scale bar = 2 μm, *n* = 4 mice per group. (**B**, **C**) JC-1 fluorescent probe was used to detect the MMP of primary astrocytes pre-incubated with celastrol (1.0 μM or 2.0 μM) and KN-93 (10 μM) for 6 h, followed by a 24-hour exposure with VCR (3 nM). *n* = 3 independent experiments. (**D**, **E**) The analysis of the ROS assay by flowcytometry in primary astrocytes pre-incubated with celastrol (1.0 μM or 2.0 μM) and KN-93 (10 μM) for 6 h, followed by a 24-hour exposure with VCR (3 nM). *n* = 3 independent experiments. Data are presented as mean ± SEM and *P < 0.05, **P < 0.01, and ***P < 0.001. Two-way ANOVA tests were performed in **C** and **E**.

The CaMKII signaling pathway is also associated with neuronal oxidative stress and apoptosis [7, 25]. We then conducted experiments to validate the inhibitory effect of celastrol on VCR-induced oxidative stress. TEM analysis revealed that in the VCR model group, axonal mitochondria (indicated by red arrow) of unmyelinated nerve fibers exhibited moderate swelling, and thinning of the mitochondrial matrix, presenting with vacuolar changes (Fig. **5A**). Compared with the VCR model group, celastrol treatment at three different doses significantly reduced the incidence of mitochondrial abnormalities, with the high-dose group showing notable improvements in mitochondrial swelling and vacuolation (Fig. **5A**). Furthermore, we assessed the MMP in primary astrocytes using JC-1 staining, and found that VCR exposure decreased MMP, which was reversed by celastrol (2 μM) and KN-93 (Figs. **5B** and **C**). These results indicated that celastrol could ameliorate VCR-induced mitochondrial damage in astrocytes. Enhanced mitochondrial oxidative stress is a primary cause of the damage observed. We measured the levels of reactive ROS in primary astrocytes and found that VCR led to an increase in ROS production, which was significantly ameliorated by both celastrol and KN-93 (Figs. **5D** and **E**). These data suggest that celastrol protects astrocytes from VCR-induced oxidative stress and associated mitochondrial damage in mice by inhibiting the CaMKII signaling pathway.

### 3.6 Celastrol Mitigates VCR-induced Apoptosis in Astrocytes

**Fig. (6):**
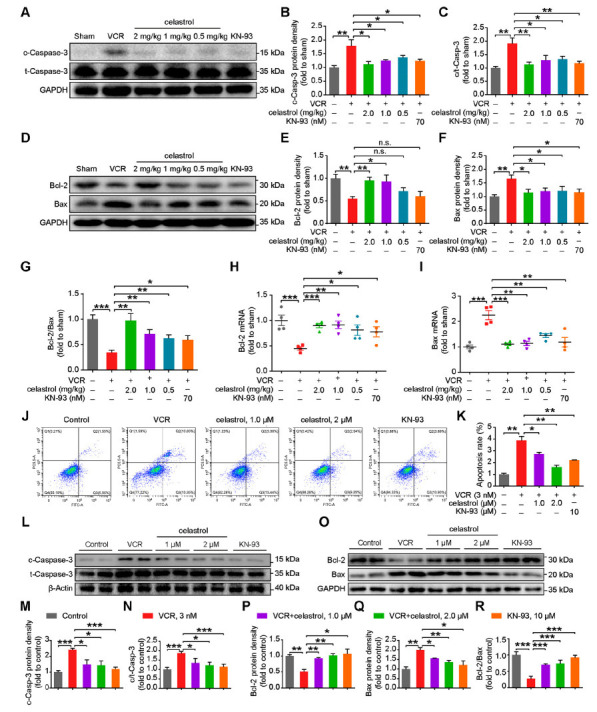
Celastrol mitigates VCR-induced apoptosis by inhibiting CaMKII phosphorylation. (**A**) Western blot and (**B**, **C**) quantitative analysis of Cleaved-Caspase3 and Caspase3 proteins expression in the spinal cord from the indicated male mice. *n =* 6 mice per group. (**D**) Western blot and (**E**-**G**) quantitative analysis of Bcl-2 and Bax proteins expression in the spinal cord from the indicated male mice. *n =* 4 mice per group. (**H**, **I**) qRT-PCR analysis of spinal cord *Bcl-2* and *Bax* mRNA levels, *n* = 4 mice/group. (**J**) Representative flow cytometry analysis of Annexin V and PI staining, and (**K**) quantitative analysis of apoptosis proportion by flowcytometry in primary astrocytes pre-incubated with celastrol (1.0 μM or 2.0 μM) and KN-93 (10 μM) for 6 h, followed by a 24-hour exposure with VCR (3 nM). *n* = 3 independent experiments. (**L**) Western blot and (**M**, **N**) quantitative analysis of Cleaved-Caspase3 and Caspase3 proteins expression in astrocytes pre-incubated with celastrol (1.0 μM or 2.0 μM) and KN-93 (10 μM) for 6 h, followed by a 24-hour exposure with VCR (3 nM). *n* = 3 independent experiments. (**O**) Western blot and (**P**-**R**) quantitative analysis of Bcl-2 and Bax proteins expression in astrocytes pre-incubated with celastrol (1.0 μM or 2.0 μM) and KN-93 (10 μM) for 6 h, followed by a 24-hour exposure with VCR (3 nM). *n* = 3 independent experiments. Data are presented as mean ± SEM and *P < 0.05, **P < 0.01, and ***P < 0.001. Unpaired two-tailed Student’s t-tests were performed in **B**, **C**, **E**-**I**, **K**, **M**, **N**, and **P**-**R**.

We then investigated the potential of celastrol to ameliorate VCR-induced apoptosis. We detected the expression levels of apoptosis-related proteins Cleaved Caspase-3, Bcl-2, and Bax in spinal cord tissues *via* Western blotting. It was found that celastrol and KN-93 significantly reversed the VCR-induced dysregulation of pro-apoptotic proteins Cleaved Caspase-3 and Bax. Notably, although the medium and high doses of celastrol and KN-93 significantly improved the expression of the anti-apoptotic protein Bcl-2, all three doses of celastrol significantly increased the Bcl-2/Bax ratio (Figs. **6A**-**G**). In alignment with these findings, qRT-PCR analysis confirmed that celastrol and KN-93 were effective in restoring the mRNA expression levels of Bcl-2 and Bax altered by VCR treatment (Figs. **6H** and **I**). Subsequently, we cultured primary astrocytes to verify the impact of celastrol on VCR-induced apoptosis *in vitro*. Flow cytometry results indicated that celastrol and KN-93 significantly inhibited the increase in apoptosis triggered by VCR (Figs. **6J** and **K**). Western blot analysis revealed that VCR treatment increased the expression of Cleaved Caspase-3 and Bax proteins while decreasing Bcl-2 protein expression in primary astrocytes. Celastrol and KN-93 markedly reversed these effects (Figs. **6L**-**R**). In summary, celastrol ameliorates VCR-induced apoptosis by inhibiting CaMKII phosphorylation.

## 4 DISCUSSION

CINP is a prevalent side effect of oncological treatment [11]. However, there is an extreme lack of medications for the treatment of CINP. In this study, VCR was employed as a model chemotherapeutic agent to induce neuropathic pain, and we investigated the therapeutic efficacy and underlying mechanisms of celastrol in VINP. We found that celastrol, in a dose-dependent manner, elevated pain thresholds and nociceptive thresholds in mice and ameliorated VCR-induced neuropathy. Concurrently, celastrol suppressed the overactivation of astrocytes and mitigated inflammation, oxidative stress, and apoptosis in the spinal dorsal horn caused by VCR through the inhibition of CaMKII phosphorylation.

Numerous studies have confirmed that astrocytes in the central nervous system play a pivotal role in the development and maintenance of chronic pain [26-28]. Peripheral nerve damage induced by chemotherapy may lead to an increase in [Ca^2+^]_i_ in spinal dorsal horn astrocytes, subsequently activating these cells [7, 23, 24]. Our research also found that celastrol inhibited astrocyte activation induced by VCR and reduced [Ca^2+^]_i_. Subsequently, we conducted a preliminary exploration of the mechanism of celastrol based on Ca^2+^ and discovered that the CaMKII protein, which is regulated by Ca^2+^ and associated with VINP, was significantly activated after VCR treatment. Celastrol can reverse this effect. Concurrently, intrathecal injection of the CaMKII phosphorylation inhibitor KN-93 significantly improved VINP. Therefore, we propose that celastrol exerts its therapeutic effect on VINP by inhibiting the Ca^2+^-dependent CaMKII activation. It should be noted that, in this study, we found that celastrol only inhibits the release of intracellular Ca^2+^ stores induced by VCR, while the influx of extracellular Ca^2+^ ions induced by VCR may also lead to the activation of astrocytes. Thus, further exploration is needed to comprehensively inhibit the increase of [Ca^2+^]_i_ caused by VCR.

Astrocyte activation in VINP is marked by cellular hypertrophy and elevated levels of GFAP, potentially associated with the generation of inflammatory mediators [21, 22]. Activated astrocytes also contribute to the progression of chemotherapy-induced peripheral neuropathy through the secretion of regulatory factors, including chemokines, pro-inflammatory/anti-inflammatory cytokines, and ATP [22, 23]. Our study identified that celastrol reduces the expression of the p-P65 protein, a critical component of the NF-κB pathway. KN-93 also provides comparable anti-inflammatory benefits by ameliorating Cx43-dependent inflammatory responses in VINP. Collectively, these findings suggest that celastrol may mediate its anti-inflammatory effects in VINP by targeting the CaMKII/Cx43 signaling pathway.

The connection between ROS and [Ca^2+^]_i_ in triggering neuronal apoptosis signaling pathways has been reported [29-31]. Oxidative stress compromises mitochondrial function, consequently disrupting energy metabolism [29-31]. Our earlier experiment has demonstrated that VCR perturbs the balance of apoptosis-associated proteins, including Cleaved Caspase-3, Bcl-2, and Bax, and increases the rate of apoptosis [7]. Additionally, we observed that VCR boosts ROS production within astrocyte mitochondria and reduces MMP, a critical indicator of mitochondrial integrity, leading to mitochondrial dysfunction. Encouragingly, this study confirmed that celastrol and KN-93 significantly mitigated VCR-induced ROS generation, restored MMP, and decreased apoptotic rates. Drawing from these observations, we propose that celastrol regulates oxidative stress within astrocyte mitochondria by suppressing CaMKII phosphorylation, thus inhibiting VCR-induced apoptosis.

Currently, first-line drugs for treating CINP, such as mirogabalin, gabapentin, and pregabalin, primarily reduce neuronal hyperexcitability by binding to the α2δ subunit of voltage-gated calcium channels [32-35]. Although these drugs demonstrate some effectiveness in symptom management, their mechanisms mainly focus on regulating nerve conduction and fail to address the underlying neuroinflammation or oxidative damage that drives the progression of CINP [34]. Celastrol can alleviate VINP by reducing astrocyte-mediated neuroinflammation, oxidative stress, and apoptosis. A key advantage of celastrol is its ability to simultaneously target multiple pathological pathways associated with CINP, distinguishing it from traditional treatment choices. This dual action not only alleviates pain but also prevents disease progression by rescuing the viability of astrocytes in the spinal cord. Additionally, the multitarget effect of celastrol indicates potential synergistic interactions with existing therapies. For example, combining low-dose gabapentin with celastrol can simultaneously block nociceptive signaling (through calcium channel modulation) and reduce neuroinflammation/oxidative stress, thereby enhancing therapeutic efficacy while reducing dose-dependent side effects (such as dizziness and sedation) [35].

Although this study provides compelling evidence for the role of celastrol in treating VINP, several issues still need to be considered. Sex differences are an important factor in the study of neuropathic pain. As emphasized by Stockstill *et al*. in their study, there are significant differences between male and female mice in neuropathic pain models [12]. These differences may be closely related to factors such as pain tolerance and hormone secretion in female mice. The present study mainly focused on male mice, lacking in-depth research on the intervention effects and mechanisms of celastrol with female mice. In our future research, we will further evaluate the protective and regulatory effects of celastrol on VINP in female mice from multiple perspectives, including pain perception and physiological mechanism regulation. Additionally, this study employed a sham group to account for procedural effects. The absence of a naïve group limits the enhancement of the interpretation of baseline physiological parameters. Future studies will include a naïve group to establish absolute baseline values.

## CONCLUSION

In this study, we identified that celastrol significantly alleviates VCR-induced overactivation of astrocytes, inflammation, oxidative stress, and apoptosis in the spinal cord by inhibiting CaMKII phosphorylation, thereby effectively mitigating VINP. As a neuroprotective agent, celastrol shows significant potential for treating CINP. Our study provides preclinical evidence of celastrol's neuroprotective effects and offers hope for new treatment options for patients with CINP.

## AUTHORS’ CONTRIBUTIONS

The authors confirm their contribution to the paper as follows: Y.H.H., Y.M.L., and G.Z.L. designed the experiments. G.Z.L. and J.X. conducted experiments and analyzed data. Y.H.H. and G.Z.L. wrote the manuscript. All authors reviewed the results and approved the final version of the manuscript.

## Figures and Tables

**Table 1:** Sequences of the primers for qRT-PCR.

Gene	Primer Sequence (5’-3’)
Mouse TNF-α	F: TCCCCAAAGGGATGAGAAG R: CACTTGGTGGTTTGCTACGA
Mouse Cox-2	F: CATCCCCTTCCTGCGAAGTT R: GGCCCTGGTGTAGTAGGAGA
Mouse Cx43	F: GGTGATGAACAGTCTGCCTTTCG R: GTGAGCCAAGTACAGGAGTGTG
Mouse Bax	F: TGGAGATGAACTGGACAGCAATAT R: GCAAAGTAGAAGAGGGCAACCAC
Mouse Bcl-2	F: CTCAGGCTGGAAGGAGAAGAT R: AAGCTGTCACAGAGGGGCTAC
Mouse GAPDH	F: AGGTCGGTGTGAACGGATTTG R: TGTAGACCATGTAGTTGAGGTCA

## Data Availability

The data and supportive information are available within the article.

## LIST OF ABBREVIATIONS

CCD: Charge-coupled Device
CINP: Chemotherapy-induced Neuropathic Pain
FBS: Fetal Bovine Serum
GFAP: Glial Fibrillary Acidic Protein
MMP: Mitochondrial Membrane Potential
PWL: Paw Withdrawal Latency
PWT: Paw Withdrawal Threshold
ROS: Reactive Oxygen Species
TNF-α: Tumor Necrosis Factor-alpha
VCR: Vincristine
VINP: Vincristine-induced Neuropathic Pain
