# Supplementary material for: Celastrol Ameliorates Vincristine-induced Neuropathic Pain by Inhibiting Spinal Astrocyte Hyperactivation-mediated Inflammation, Oxidative Stress, and Apoptosis
Source: Curr Neuropharmacol. 2025 May 14;24(3):419–32. doi: 10.2174/011570159X385690250509050208 (PMC13084761; doi:10.2174/011570159X385690250509050208)
Supplement: Supplementary file 1 [file CN-24-3-419_SD1.pdf]

## Supplementary Material

# Celastrol Ameliorates Vincristine-induced Neuropathic Pain by Inhibiting Spinal Astrocyte Hyperactivation-mediated Inflammation, Oxidative Stress, and Apoptosis

Gui-Zhou Li[1,2,3], Jing Xu[2], Yun-Man Li[3] and Ya-Hui Hu[2,*]

*[1]Ministry of Education Key Laboratory of Model Animal for Disease Study, Model Animal Research Center, Jiangsu Key Laboratory of Molecular Medicine, Medical School, Nanjing University, Nanjing 210032, China; [2]Pharmaceutical Sciences Research Center, Department of Pharmacy, Children's Hospital of Nanjing Medical University, Nanjing 210009, China; [3]School of Basic Medicine and Clinical Pharmacy, China Pharmaceutical University, 24 Tongjiaxiang, Nanjing 210009, China*

**1875-6190/26 $65.00+.00** **© 2026 Bentham Science Publishers**

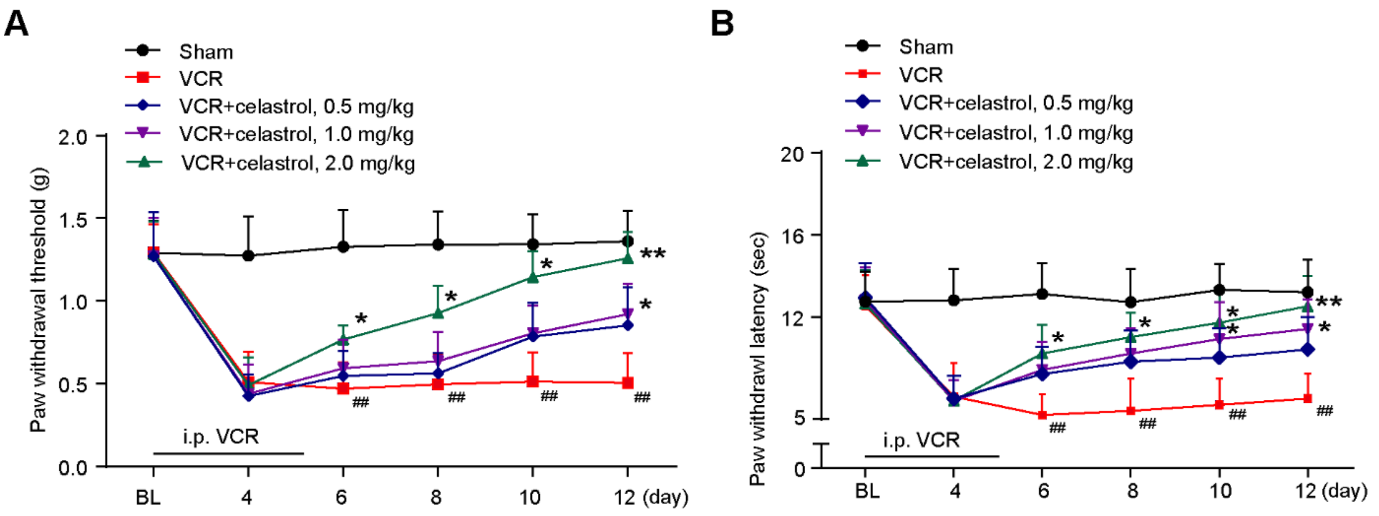


**Fig. (S1). Celastrol Alleviates VINP and Sciatic Nerve Injury in Mice.** Celastrol attenuated mechanical (**A**) and thermal hyperalgesia (**B**) in female mice with VINP. Celastrol (0.5, 1 and 2 mg/kg, i.p.) was administered after the VCR (0.1 mg/kg) intraperitoneal injection. $n = 8$ mice per group. Data are presented as mean ± SEM. Sham *vs.*VCR: ##, and VCR *vs.*VCR+celastrol:*. #*P* < 0.05, ##*P* < 0.01, and ###*P* < 0.001. **P* < 0.05, ***P* < 0.01, and ****P* < 0.001. Two-way ANOVA tests were performed in **A** and **B.**

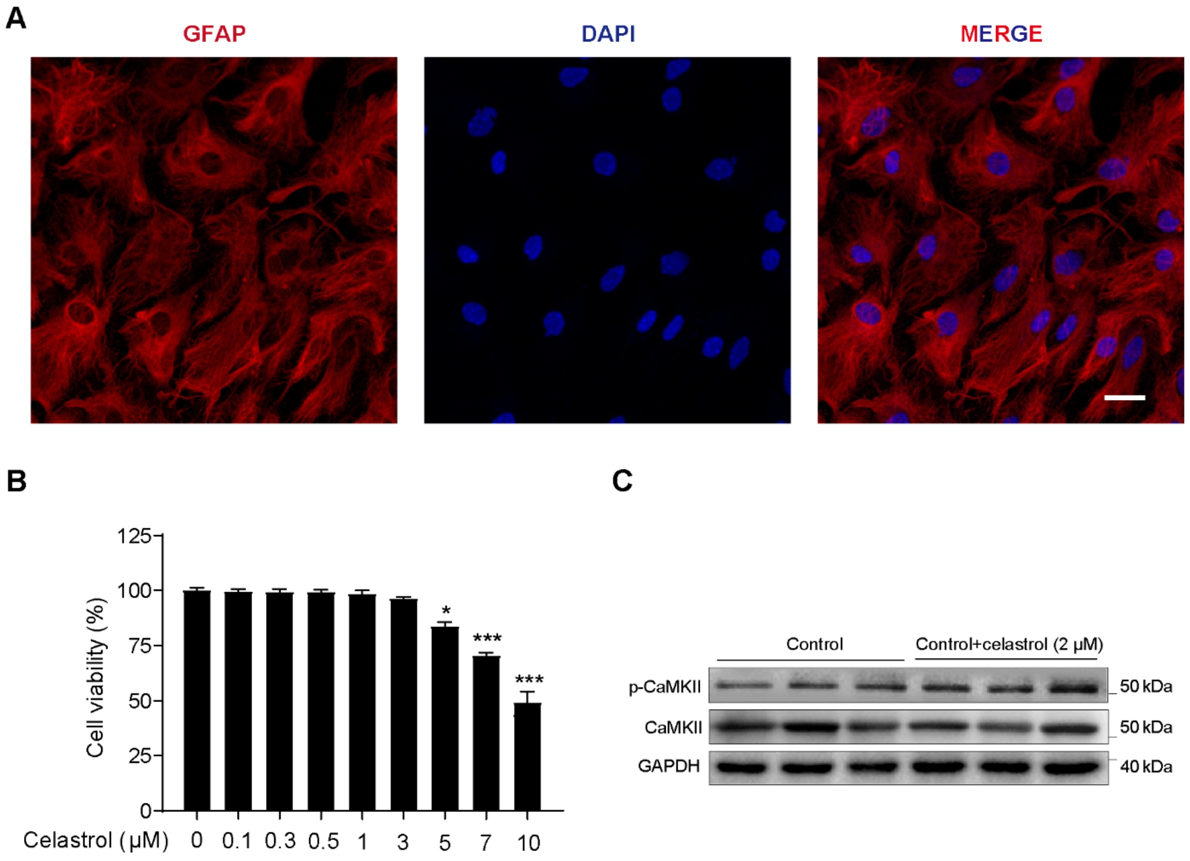


**Fig. (S2). The effect of celastrol on astrocytes.** (**A**) Representative confocal images of GFAP (red) and DAPI (blue). scale bar = 100 μm, $n$ = 3 independent experiments. (**B**) Cell viability of astrocytes pre-incubated with varying concentrations of celastrol from 0.1 to 10 μM for 6 h. $n$ = 6 cultures/group. (**C**) Western blot analysis of CaMKII and p-CaMKII protein expression in astrocytes pre-incubated with celastrol (2.0 μM) for 6 h. $n$ = 3 independent experiments. Data are presented as mean ± SEM and **P* < 0.05, ***P* < 0.01, and ****P* < 0.001. Unpaired two-tailed Student's t-tests were performed in **B**.
